# Immune-Enhancing Activity of Aqueous Extracts from *Artemisia rupestris* L. via MAPK and NF-kB Pathways of TLR4/TLR2 Downstream in Dendritic Cells

**DOI:** 10.3390/vaccines8030525

**Published:** 2020-09-13

**Authors:** Yu Yang, DanYang Wang, QuanXiao Li, Jiang He, Bin Wang, Jinyao Li, Ailian Zhang

**Affiliations:** 1 Xinjiang Key Laboratory of Biological Resources and Genetic Engineering, College of Life Science and Technology, Xinjiang University, Urumqi 830046, China; shandongyangyu@163.com (Y.Y.); danyangwang_love@163.com (D.W.); liquanxiao002@163.com (Q.L.); ljyxju@xju.edu.cn (J.L.); 2 Key Laboratory of Uighur Medicine, Xinjiang Institute of Materia Medica, Xinjiang 830004, China; hj_1211@163.com; 3 Key Lab of Medical Molecular Virology, School of Basic Medical Science, Shanghai Medical College, Fudan University, Shanghai 200032, China; bwang3@fudan.edu.cn

**Keywords:** water-extractable polysaccharides, *Artemisia rupestris* L., dendritic cells, toll-like receptors, immunomodulation

## Abstract

*Artemisia rupestris* L. has long been used as a traditional herbal medicine owing to its immunomodulatory activity. Aqueous extracts of *Artemisia rupestris* L. (AEAR) contain the main functional component and can activate the maturation of dendritic cells (DCs) and enhance the adaptive immunity as the adjuvant against infections. To explore the underlying mechanism of immunomodulatory activities of AEAR, DCs were produced from bone-marrow cells of mice and the effects of AEAR on cell viability were assessed by the Cell Counting Kit 8 (CCK8) method and annexin V/propidium iodide staining assays. Then, the effects of AEAR on the morphology, maturation, and function of DCs were detected using a microscope, flow cytometry-based surface receptor characterization, and endocytosis assays. The secretion levels of cytokines were then analyzed with enzyme-linked immunosorbent assay (ELISA). The activation state of DCs was evaluated by the mixed lymphocyte reaction (MLR). The activity of MAPKs and NF-κB pathways, which were involved in the regulation of AEAR on DCs, was further detected by Western blot. AEAR did not have a cytotoxic effect on DCs or mouse splenocytes. AEAR remarkably enhanced the phenotypic maturation of DCs and promoted the expression of costimulatory molecules and the secretion of cytokines in DCs. AEAR also significantly decreased the phagocytic ability of DCs and augmented the abilities of DCs to present antigens and stimulate allogeneic T-cell proliferation. Simultaneously, AEAR potently activated toll-like receptor (TLR)4-/TLR2-related MAPKs and induced the degradation of IκB and the translocation of NF-κB. In short, AEAR can profoundly enhance the immune-modulating activities of DCs via TLR4-/TLR2-mediated activation of MAPKs and NF-κB signaling pathways and is a promising candidate immunopotentiator for vaccines.

## 1. Introduction

Vaccination can prevent and control the spread of infectious diseases. It is still needed to explore new vaccines for diseases that continue to threaten human and animal health, such as HIV, malaria, and Foot-and-mouth disease virus (FMDV) [1]. Traditional Chinese medicines (TCMs) and plants contain many active compounds such as polysaccharides, which show significant advantages in the treatment and prevention of infectious diseases, such as influenza, hepatitis B, asthma, and anthrax [2,3,4,5], thanks to their compatibility, non-toxicity, and therapeutic properties. The various biological activities of polysaccharides, such as antiviral, hypoglycaemic, and immunostimulatory activities, have been reported [6,7,8]. Among these bio-activities, the immunoregulatory activity of polysaccharides has been particularly widely concerned as they can significantly affect immune systems through immunological cell membrane receptors, and trigger several cellular events including the activation of innate immune cells and antigen-presenting cells as well as the secretion of cytokines. Polysaccharides from TCM, such as *Astragalus, lycium barbarum, Angelica,* and *inulin* [9,10,11,12], are believed to possess immunomodulatory activities. Therefore, it is feasible to screen bioactive polysaccharides derived from aqueous extracts of TCM as an adjuvant.

As a part of TCM, *Artemisia rupestris* L. (Yizhihao in Chinese) is widely distributed in Xinjiang of China, Asia, and Europe, among others, and has been proven to be safe and less toxic in animal experiments and is widely used in the treatment of influenza virus and acute or chronic inflammation [13]. It is mainly composed of polysaccharides, flavonoids, alkaloids, and other components [14]. The diverse bio-activities of *Artemisia rupestris* L. are mainly attributed to its polysaccharides. The immunomodulatory activity of *Artemisia rupestris* L. is good with low toxicity. In recent years, our studies indicated that the administration of aqueous extracts of *Artemisia rupestris* L. (AEAR), water-extractable crude polysaccharides, could induce the functional and phenotypic maturation, the activation of T lymphocytes, and the upregulation of TNF-α and IL-12 production of dendritic cells (DCs), which was a critical step in adaptive immune responses and inflammation [15]. We also found that crude polysaccharides obtained from *Artemisia rupestris* L. could enhance the adaptive immune responses of FMDV vaccine and influenza vaccine as an adjuvant [13,16]. However, the advantages and potential application of AEAR as the adjuvant in the prevention of infectious diseases should be further explored. Although the potency of AEAR in promoting the efficiency of immunomodulation has been confirmed, the exact molecular mechanism that AEAR modulates the function of DCs and immune response has not been clearly elucidated. Therefore, it is significant to further explore the functions of AEAR based on known mechanisms for the purpose of designing adjuvants against infectious diseases.

DCs as the initiator and modulator of the immune response play a vital role in the induction of immunity. DCs can control the function of B and T lymphocytes by capturing and processing antigens to activate antigen-specific T cells, and secreting immunomodulatory cytokines (IL-1β, IL-6, IL-12p70, and TNF-α) to direct the immune responses against infectious agents [17]. Recent studies demonstrated that polysaccharides from TCM modulated immune functions via DCs, a key player in the immune system. DCs’ activation is mediated primarily via several pattern recognition receptors (PRRs) [18]. Polysaccharides activate DCs through stimulating the PRRs on the surface of cells. Toll-like receptors (TLRs) are involved in the activation of DCs as well as the defense against disease infection [19]. Among TLRs, TLR4 and TLR2 play the most significant role in DCs’ activation [18,20]. Binding to TLR2 and TLR4 is the initial event in the activation of antigen-presenting cell (APC) and adaptive immunity and the downstream activation of key NF-κB transcription factors and MAPKs pathways [21,22]. The activation of TLR2 and TLR4 involves many stimuli such as lipopolysaccharide (LPS), Taxol, and polysaccharides [23,24].

In our previous study, we found that TLR4 played an important role in the activation of DCs by AEAR. Up to now, it is unknown whether TLR2 is also the receptor of AEAR. In the study, we explored the roles of TLR2 in AEAR-induced immunomodulation of DCs and related pathways.

## 2. Materials and Methods

### 2.1. Materials and Reagents

*Artemisia rupestris* L. was obtained from Xinjiang Institute of Materia Medica in Urumqi and was identified by Professor Jiang He. A voucher specimen (No. AR13020307) was deposited in the Xinjiang Key Laboratory of Biological Resources and Genetic Engineering, College of Life Science and Technology, Xinjiang University, China. AEAR was obtained from *Artemisia rupestris* L. according to the previous report [15]. Briefly, dried powder of leafs and stems was extracted in hot water at 80 °C for 4 h and then refluxed to eliminate colored components and lipids. The obtained filtrate was concentrated into one-twentieth volume and precipitated in ethanol. Then, the sediment was dissolved in water and proteins were removed by the Savage method [25]. Finally, crude polysaccharides were acquired. The results of the phenol-sulfuric acid method indicated that total sugar content reached 32.39%. Obtained AEAR was dissolved with 0.9% saline to prepare the stock solution and then stored at 4 °C for use. Before each experiment, the stock solution was diluted as required.

### 2.2. Infrared Spectroscopic Analysis

The samples were mixed with KBr and pressed into pellets to determine the infrared spectra in the range of 400–4000 cm^−1^.

### 2.3. Animals and Generation of DCs

Female C57BL/6 mice (6–8 weeks old) were from Xinjiang Medical University (Urumqi, China). The animal experiments were approved by the Committee on the Ethics of Animal Experiments of Xinjiang Key Laboratory of Biological Resources and Genetic Engineering in Xinjiang University (BRGE-AE001).

DCs were generated according to an established method. Briefly, bone marrow of the tibia and femur from Naïve C57BL/6 mice was separated. Bone marrow was flushed in culture medium, spun down (1200 rpm, 5 min), and re-suspended in RPMI-1640 medium (Gibco, Gaithersburg, MD, USA) containing 20 ng/mL GM-CSF (PeproTech, Rocky Hill, NJ, USA). After 6 days, immature bone marrow DCs (BM-DCs) were harvested for subsequent assays.

### 2.4. Cell Viability Assays

To explore the influences of AEAR on splenocytes and DCs of mice, cell viability was determined by CCK-8 kit (Biosharp, Hefei, China). DCs were acquired as described above. Splenocyte suspensions were prepared and then the lysis of erythrocytes was performed. DCs (1 × 10^5^ cells/well) or single-cell suspension of splenocytes (1 × 10^6^ cells/well) were seeded in 96-well plates and then allowed to attach the plates for 48 h after adding AEAR at serial concentrations (10 to 1600 μg/mL) in triplicate. In the positive control, LPS (Sigma-Aldrich, St. Louis, MO, USA) was added to replace AEAR. After cells were incubated at 37 °C together with CCK-8 working solution for 4 h, the supernatant was removed, followed by the addition of DMSO. Then, the absorbance at 570 nm was recorded with a microplate reader (Bio-Rad, Hercules, CA, USA). Cell survival rate was calculated as follows: (absorbance of the experimental group/absorbance of untreated control group) × 100%.

### 2.5. Morphological Observations

The morphology of DCs was observed under an inverted phase-contrast microscope (Nikon, Japan). Immature DCs (5 × 10^5^ cells/well) were cultured on a 24-well plate, and DCs were stimulated with different concentrations of AEAR (0, 10, 100, 200, and 400 μg/mL) or LPS (100 ng/mL) for 24 h. The photos (magnification = 200× and 400×) were captured.

### 2.6. Flow Cytometric Detection of Apoptotic Cells

In the apoptosis analysis, an FITC-Annexin V/PI apoptosis kit (Yeasen, Shanghai, China) was used according to the manufacturer’s instructions. DCs were added in a 24-well plate (1 × 10^6^ cells/well) in RPMI-1640 medium without or with AEAR (10 to 400 μg/mL) or LPS (100 ng/mL) in triplicate and stood for 24 h. DCs were washed and incubated with FITC-Annexin V and PI for 15 min, and washed three times with 5 mL iced PBS. The samples were analyzed immediately on a flow cytometer (BD Biosciences, San Jose, CA, USA). The apoptosis rate was calculated as follows: apoptosis rate (%) = (the number of apoptotic cells) / (the total number of observed cells) × 100%.

### 2.7. Flow Cytometric Phenotype Analysis of DCs

The phenotypic maturation of DCs was analyzed with a flow cytometer. DCs (1 × 10^6^ cells per 1 mL) were respectively stimulated with different concentrations of AEAR (10 to 400 μg/mL) or LPS for different periods (1, 6, 12, or 24 h), and then anti-CD11c, anti-CD40, and anti-CD86 (BD) antibodies were added for 30 min staining. Cells were washed to remove excessive antibodies. The expression levels of CD86 and CD40 were measured using a flow cytometer. For Polymyxin B (PMB, Sigma, St. Louis, MO, USA) experiment, different concentrations of AEAR (10, 50, and 100 μg/mL) were pretreated with or without PMB (100 μg/mL) at 37 °C for 1 h, and then used to stimulate DCs (1 × 10^6^ cells per 1 mL) for 12 h; then, after being stained with antibody, DCs were measured by a flow cytometer. The measurement data were analyzed in FlowJo.

### 2.8. ELISA Analysis of Cytokines

DCs (1 × 10^6^ cells per 1 mL) were stimulated with different concentrations of AEAR (10 to 400 μg/mL) or LPS (100 ng/mL) for 12 h. IL-6 and IL-1β were generated in the supernatant of DCs’ cultures with ELISA kits (Boster, Wuhan, China). The absorbance at 450 nm was measured using a microplate reader. The concentrations of IL-6 and IL-1β were calculated with the standard curve.

### 2.9. Endocytosis Assays

To evaluate the uptake of dextran by DCs, the cells were collected after the co-culture with AEAR (10 to 400 μg/mL) or LPS and then incubated with 25 μg/mL FITC-dextran (Sigma, USA) for 1 h at 37 °C. Then, the reaction was stopped by PBS with 3% fetal calf serum (FBS) (Hyclone) After washing three times, the cells were stained for 30 min with PE-CD11c antibody. Non-specific binding of FITC-dextran to DCs was determined at 4 °C in parallel experiments. The double-stained DCs were analyzed using a flow cytometer.

### 2.10. Mixed Lymphocyte Reaction

MLR was utilized to evaluate the allogeneic stimulatory activity by the MTT (Sigma, USA) method. The spleens from BALB/c mice were removed aseptically and then added in RPMI-1640 medium. Mature DCs from C57BL/6 (matured in the presence of AEAR or LPS) were pretreated with 100 ng/mL mitomycin C (Sigma) at 37 °C for 30 min. After carefully washing, DCs were mixed with splenocytes according to a ratio of 1:5 (DCs/splenocytes = 4 × 10^4^: 2 × 10^5^) or 1:10 (DCs/splenocytes = 2 × 10^4^: 2 × 10^5^) in 96-well plates and then stood for 2 days. LPS was added in the positive control. Finally, the absorbances at 490 nm/630 nm were determined with a microtiter plate reader as the index of DCs stimulating the proliferation of T cells.

### 2.11. Blocking of TLR Pathways with Monoclonal Antibodies

To determine whether TLR2 or TLR4 was involved in the AEAR-induced DCs activation, DCs were pre-incubated with anti-TLR2 (5 μg/mL) or anti-TLR4 (2 μg/mL) (Novus Biologicals, Littleton, CO, USA) for 1 h, and then AEAR (50 or 400 μg/mL) was added for 12 h incubation. The expression levels of CD40, CD86, IL-1β, and IL-6 were measured as described above.

### 2.12. Western Blotting Analysis

To clarify the roles of MAPK and NF-κB pathways in AEAR-stimulated DCs, DCs were incubated together with 200 μg/mL AEAR for different periods (0, 5, 10, 20, 30, 45, 60, and 120 min). After AEAR treatment, DCs were collected and proteins were extracted with the nuclear and cytoplasmic extraction kit (Cwbiotech, Beijing, China). The protein concentration was then determined by BCA Protein Assay kit (Cwbiotech, Beijing, China). After boiling, the samples from different AEAR samples were separated by 12% SDS-PAGE and then transferred to PVDF blotting membranes. After blocking with PBS containing 5% skimmed milk for 1 h, membranes were incubated overnight at 4 °C with appropriately diluted primary antibodies against cytoplasmic non-modified and phosphorylated proteins (ERK, p-ERK, JNK, NF-κB-p65, p-JNK, p38, p-p38, p-IκB-α, IκB-α, IKK-α, IKK-β, p-IKK-α/β, and p-NF-κB-p65) (Cell Signaling Technology, Beverly, MA, USA). Membranes were incubated with secondary antibodies for 1 h. Signals were captured with the enhanced chemiluminescence (ECL) kit.

### 2.13. Data Analysis

Experimental data are expressed as mean ± standard deviation (S.D.). Statistical differences between experimental groups were tested with GraphPad Prism 5.0 through one-way analysis of variance (ANOVA) and Tukey’s multiple-comparison test. *p* < 0.05 was considered to be significant.

## 3. Results

### 3.1. FT-IR Spectral Features of AEAR

**Figure 1:**
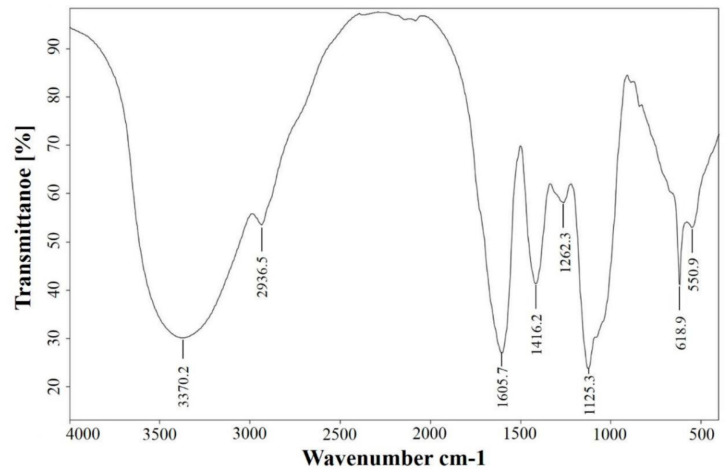
Infrared spectral analysis of *Artemisia rupestris* L. (AEAR).

FT-IR spectra of AEAR are shown in Figure 1. The peaks at 3370.19 cm^−1^ and at 1605.68 cm^−1^ were ascribed to the stretching vibration of -OH and aldehyde group (–CHO), respectively. The peak at 1416.25 cm^−1^ could be ascribed to O-H or C-H bending and C-O stretching. The peak at 1125.1 cm^−1^ was ascribed to the asymmetric vibration of the C–O–C glycosidic bond.

### 3.2. Effects of AEAR on Cytotoxicity

**Figure 2:**
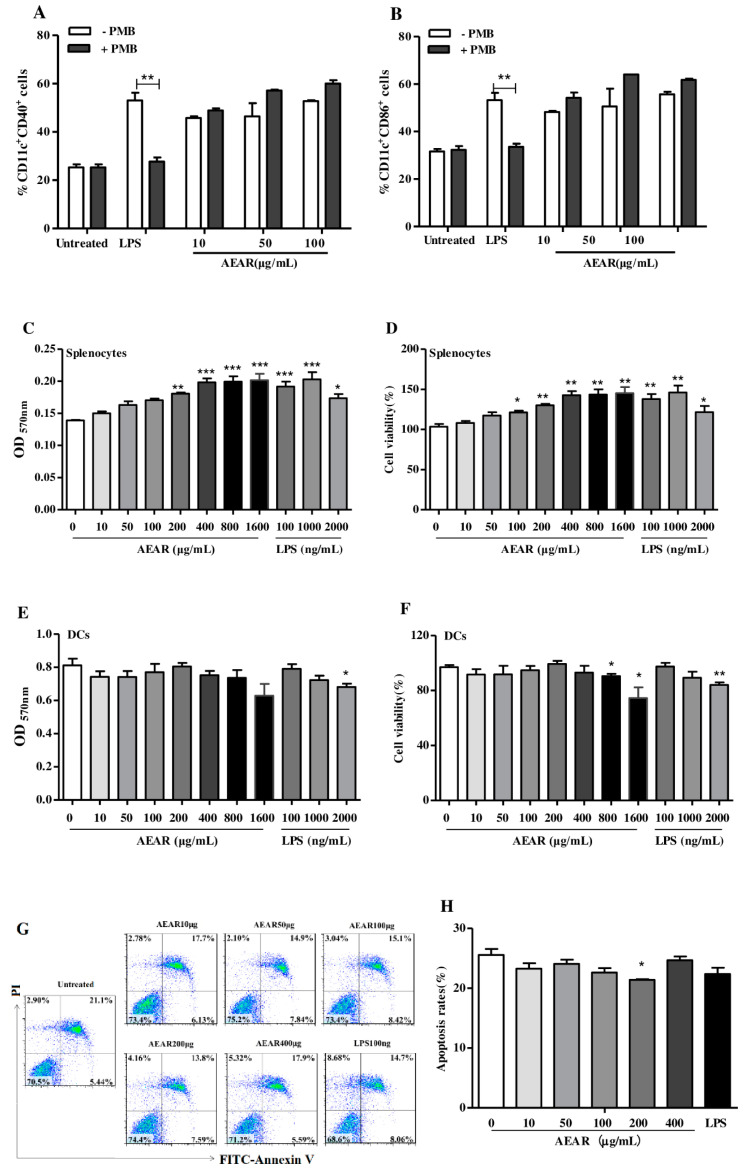
Effects of AEAR on the viability. (**A**,**B**) BM-dendritic cells (DCs) were induced under the existence of GM-CSF for 6 days and AEAR (10, 50, and 100 μg/mL) or LPS (100 ng/mL) were pretreated with or without PMB (100 μg/mL) for 1 h, and then used to stimulate DCs (1 × 10^6^ per 1 mL); FACS detected the level of CD40 and CD86 on DCs. (**C**–**F**) Splenocytes and DCs were incubated with AEAR and the cell viability was measured with a CCK-8 kit. (**G**,**H**) DCs were stimulated and then stained with Annexin V/PI for apoptosis analysis. Late apoptotic cells marked by Annexin V/PI were in the upper right quadrant, whereas early apoptotic cells marked by Annexin V/PI were in the lower right quadrant. Representative dot plots showed the distribution of different cell populations and the percentage of apoptosis. The values are expressed as mean ± SD (n = 3). In A–B, ** indicates the significant difference between LPS and LPS pretreated with PMB at *p* < 0.01. In C–H, * *p* < 0.05, ** *p* < 0.01 and *** *p* < 0.001 indicate significant and extremely significant differences, respectively, compared with the untreated DCs group, determined by one-way analysis of variance (ANOVA) and Tukey’s multiple-comparison test.

LPS, the most common endotoxin, is a known immunomodulator and often a contaminant in biological preparations. PMB is a cationic polypeptide anti-biotic with a high affinity for the lipid A component of LPS, which results in neutralization of endotoxin-like bioactivity of most forms of LPS. To ensure that the effect of AEAR was not due to endotoxin contamination, as shown in Figure 2A,B, no difference was observed in CD40 and CD86 expression in DCs treated with AEAR in the absence or presence of PMB. However, a dramatic decline of CD40 and D80 expression was observed in LPS-induced DCs in the presence of PMB. These results demonstrated that AEAR was free of LPS contamination.

With the MTT method, the potential cytotoxicity of AEAR on splenocytes and DCs was determined. The cells were stimulated with AEAR for 48 h and the cell viability was detected through CCK-8 assays (Figure 2C,D). Compared with the results of the control groups, AEAR (10 to 1600 μg/mL) or LPS (100 to 2000 μg/mL) in the experimental groups was not cytotoxic to splenocytes. In contrast, the cell viability of splenocytes showed a significant proliferation in a dose-dependent manner (*p* < 0.05) and was nearly increased by 45% in the AEAR concentration range of 400–1600 μg/mL. Similar results were obtained in DCs (Figure 2E,F). AEAR with the concentration range of 10 to 400 μg/mL was not cytotoxic and displayed mild growth inhibition activity. Cell viability was decreased by 7% under 800 μg/mL AEAR. However, 1600 μg/mL AEAR showed a significant cytotoxic effect against DCs (*p* < 0.05). Thus, in the subsequent experiments, the AEAR concentration range was from 10 to 400 μg/mL.

To further observe the inhibitory effect of AEAR on DCs’ growth, the apoptosis analysis was performed. After DCs were stimulated with different concentrations of AEAR and LPS for 24 h, Annexin V/PI staining was conducted (Figure 2G,H). The results showed that AEAR could significantly decrease the apoptosis rate of DCs. AEAR was not toxic to DCs when the AEAR concentration was below 400 μg/mL.

### 3.3. Effects of AEAR on Morphological Changes of Cells

**Figure 3:**
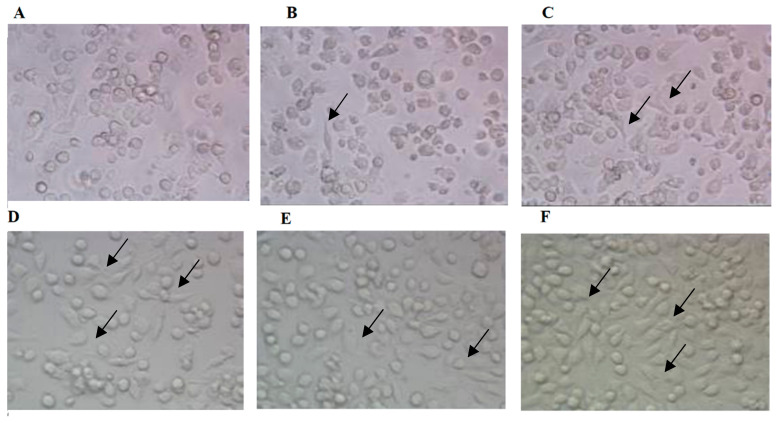
Morphological changes of DCs stimulated by AEAR. Cells were treated with 0 μg/mL (**A**), 10 μg/mL (**B**), 100 μg/mL (**C**), 200 μg/mL (**D**), and 400 μg/mL (**E**) AEAR or 100 ng/mL LPS (**F**) for 24 h. The morphological changes were observed under an inverted microscope (400×).

To observe the effects of AEAR on DCs’ maturation in terms of morphology, immature DCs were cultured for 6 d in the presence of GM-CSF and then stimulated for 24 h with AEAR or LPS. Then, the cells were observed under an inverted microscope (Figure 3). Mature DCs stimulated by AEAR and LPS were larger cells with an irregular or oval shape, whereas untreated DCs showed no obvious mature change. The difference suggested that AEAR stimulated the maturation of DCs.

### 3.4. AEAR Induced DCs’ Activation

**Figure 4:**
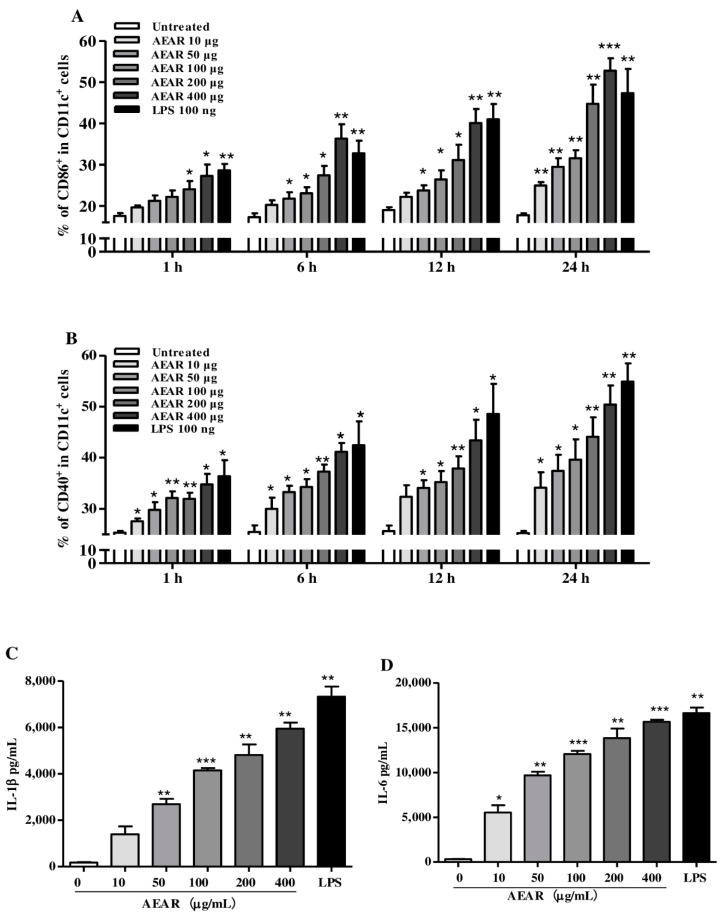
AEAR induced DCs’ activation. (**A**,**B**) Immature DCs were treated with AEAR or LPS for different periods. DCs were harvested and then stained with anti-CD11c, anti-CD40, and anti-CD86 antibodies. The expression levels of CD40 and CD86 were analyzed with a flow cytometer. (**C**,**D**) The expression levels of IL-1β and IL-6 in culture supernatants were analyzed by ELISA kit. Data were expressed as means ± SD (n = 3). * *p* < 0.05, ** *p* < 0.01, and *** *p* < 0.001 indicate significant differences compared with the untreated control, determined by one-way analysis of variance (ANOVA) and Tukey’s multiple-comparison test.

DCs’ activation is critical for inducing immune responses and can be characterized by costimulatory molecule expression and cytokines. Therefore, the expression levels of CD86 and CD40 in DCs were determined (Figure 4A,B). The expression levels of CD40 and CD86 were increased significantly after the stimulation with AEAR in a time-dependent and dose-dependent manner (*p* < 0.05). The expression levels of IL-1β and IL-6 increased significantly in a dose-dependent manner in the groups of AEAR treatment and LPS (100 ng/mL) stimulation compared with the blank control (*p* < 0.05, Figure 4C,D). Experimental data demonstrated that AEAR was an effective immunostimulator of DCs’ maturation.

### 3.5. Effects of AEAR on Endocytosis Activity of DCs

**Figure 5:**
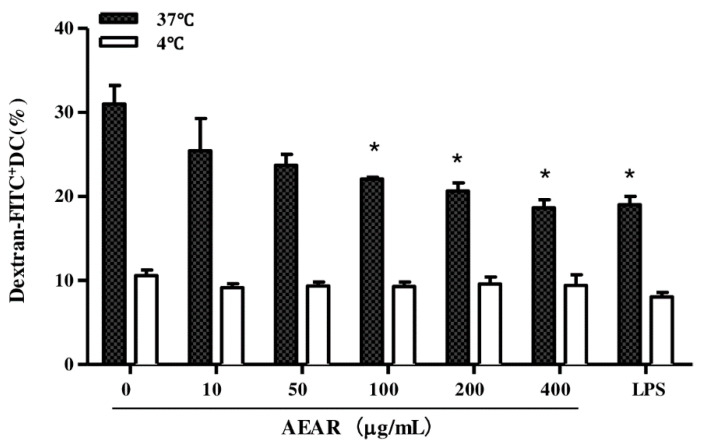
Effects of AEAR on the endocytosis activity of DCs. DCs were treated with AEAR or LPS for 12 h and then incubated with FITC-dextran for 1 h at 37 °C and 4 °C. The cells were collected to detect the phagocytic activity. Data are expressed as mean ± SD (n = 3). * *p* < 0.05 indicates significant differences compared with the untreated DCs group, determined by one-way analysis of variance (ANOVA) and Tukey’s multiple-comparison test.

The phagocytic capability was decreased after immature DCs captured antigens [26,27]. The endocytosis of DCs treated with AEAR was examined based the monitored uptake data of FITC-dextran (Figure 5). Compared with the high level of endocytosis in the untreated group, the endocytic ability of DCs treated with different concentrations of AEAR (100, 200, and 400 μg/mL) was significantly decreased (*p* < 0.05), indicating that AEAR significantly affected DCs’ maturation.

### 3.6. AEAR-Treated DCs Enhanced Splenocyte Proliferation 

**Figure 6:**
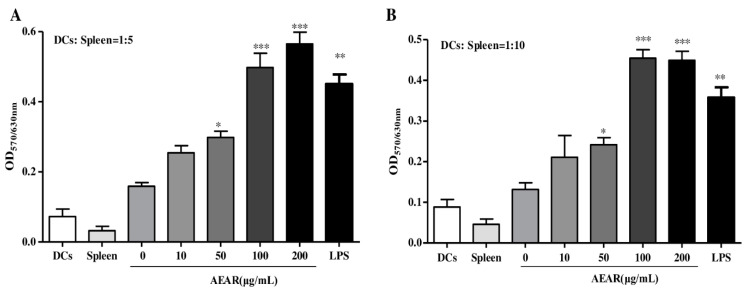
Allogeneic T cells’ activation by AEAR-treated DCs. Allogeneic splenocytes were co-cultured for 2 days according to the indicated ratio with DCs, which had been cultured for 12 h. MTT was added into the culture for the final 4 h treatment and T cell proliferation was then determined by ELISA. (**A**) DCs were mixed with splenocytes according to a ratio of 1:5. (**B**) DCs were mixed with splenocytes according to a ratio of 1:10. Data are expressed as mean ± SD (n = 3). * *p* < 0.05, ** *p* < 0.01, and *** *p* < 0.001 indicate significant differences compared with the untreated DCs group, determined by one-way analysis of variance (ANOVA) and Tukey’s multiple-comparison test.

Compared with immature DCs, mature DCs could more significantly stimulate the proliferation of allogeneic T cells [28]. The effects of AEAR on allogeneic lymphocyte activation by DCs were further explored through MLR reaction (Figure 6). AEAR-stimulated DCs from C57BL/6 mice were mixed together with splenocytes from BALB/c mice for 48 h according to the ratios of 1:5 and 1:10. AEAR-treated DCs could significantly enhance allogeneic splenocyte proliferation compared with the untreated DCs group (*p* < 0.05). The difference suggested that AEAR-treated DCs stimulated allogeneic T cell proliferation.

### 3.7. TLR2 Mediated DCs’ Activation by AEAR

**Figure 7:**
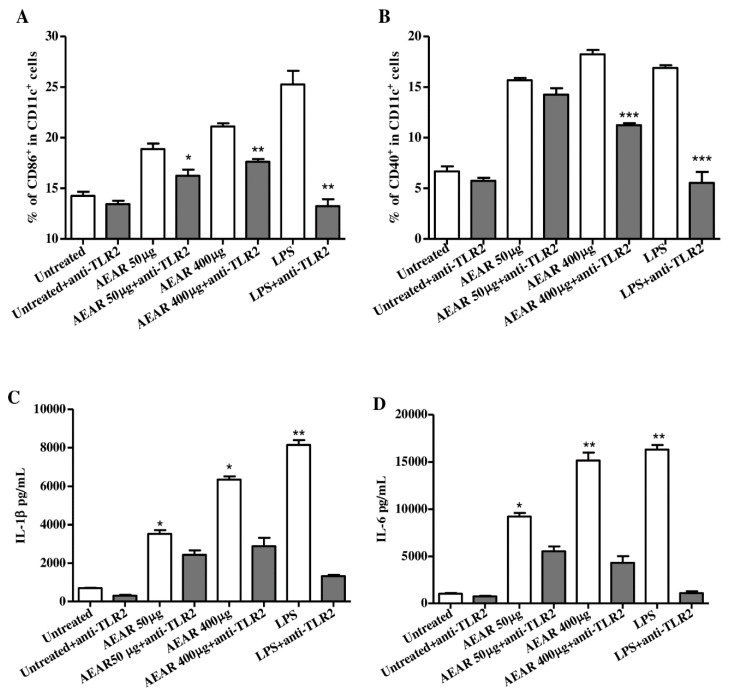
TLR2-dependent activation of DCs by AEAR. The activation of DCs was blocked by anti-TLR2 antibody. (**A**,**B**) Expression levels of CD86 and CD40. (**C**,**D**) Production of IL-1β and IL-6. The values are expressed as means ± SD (n = 3). * *p* < 0.05, ** *p* < 0.01, and *** *p* < 0.001 indicate significant differences compared with the group treated by AEAR alone, determined by one-way analysis of variance (ANOVA) and Tukey’s multiple-comparison test.

To explore the activation mechanism and investigate the membrane receptor involved in DCs’ activation and cytokine production, the interaction between TLR2 and AEAR was blocked with anti-TLR2 antibody and the influence of TLR2 on AEAR-induced DCs’ activation was evaluated in terms of cell surface molecules in DCs (Figure 7A,B) and cytokines (Figure 7C,D). It was found that the pretreatment of DCs with anti-TLR2 antibody remarkably inhibited the expressions of CD86 and CD40 (*p* < 0.05). Similarly, the yields of IL-1β and IL-6 were also significantly suppressed by AEAR treatment (*p* < 0.05), suggesting that AEAR induced DCs’ activation and cytokine production via TLR2.

### 3.8. TLR4 Mediated DCs Activation by AEAR

**Figure 8:**
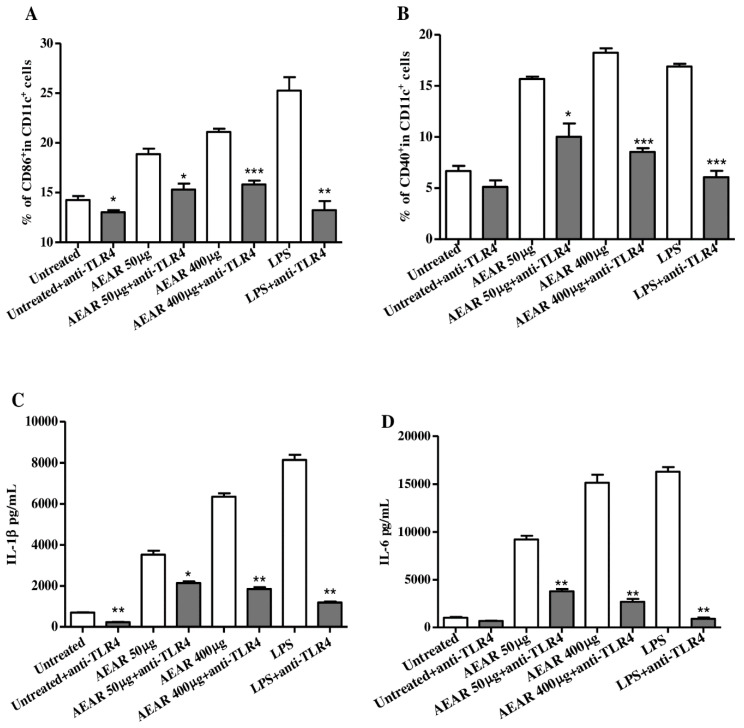
TLR4-dependent activation of DCs by AEAR. The activation of DCs was blocked by anti-TLR4 antibody. (**A**,**B**) Expression levels of CD86 and CD40. (**C**,**D**) Production of IL-1β and IL-6. The values are expressed as means ± SD (n = 3). * *p* < 0.05, ** *p* < 0.01, and *** *p* < 0.001 indicate significant differences compared with the group treated by AEAR alone, determined by one-way analysis of variance (ANOVA) and Tukey’s multiple-comparison test.

To determine whether TLR4 was also involved in the AEAR-induced activation of DCs, the collected cells and culture supernatants of DCs were analyzed after the treatment with anti-TLR4 antibody (Figure 8). The up-regulation of CD86 and CD40 induced by AEAR was significantly inhibited by blocking TLR4 pathways to different degrees (*p* < 0.05). The yields of IL-1β and IL-6 were decreased significantly by anti-TLR4 antibody (*p* < 0.05). The differences suggested that TLR4 might participate in signaling pathways of AEAR-treated DCs.

### 3.9. Effects of AEAR on the Activation of NF-kB and MAPKs in DCs

**Figure 9:**
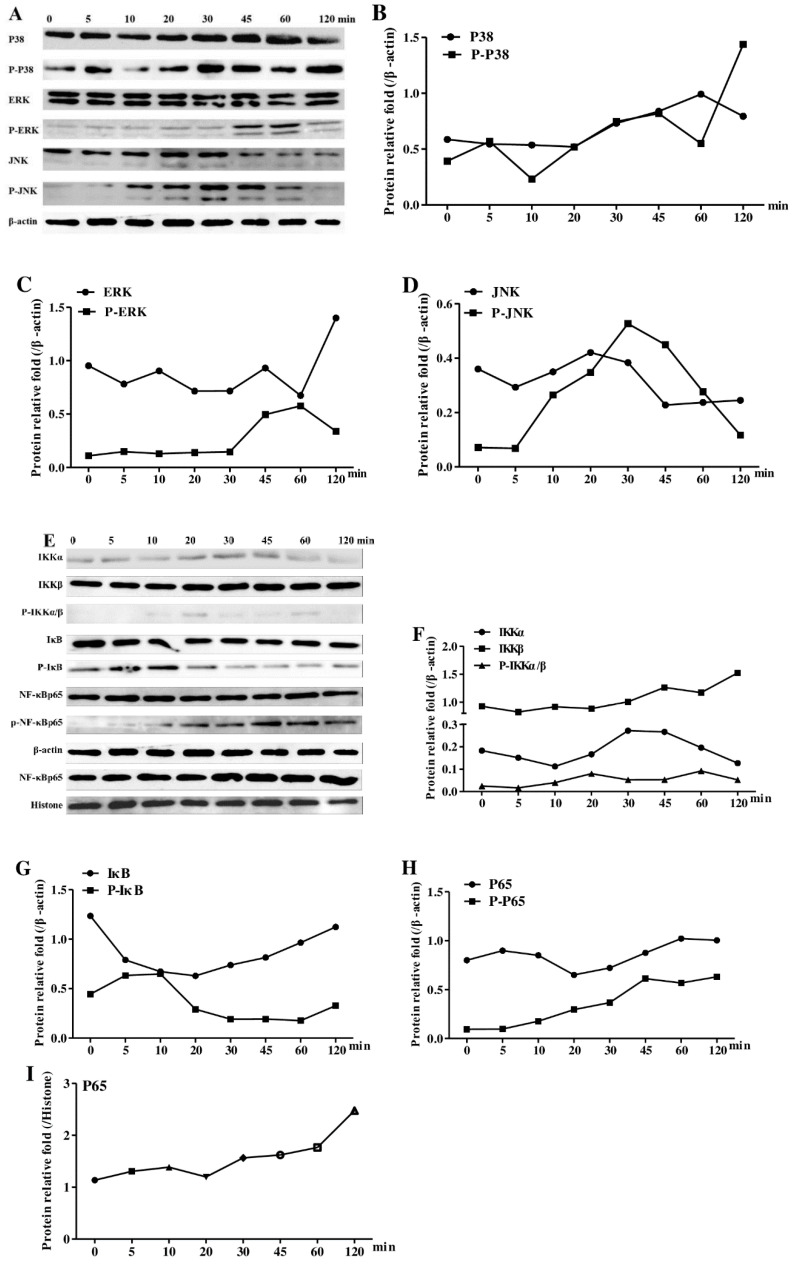
Effect of AEAR on MAPK and NF-κB signaling pathway in DCs. Immature DCs treated with AEAR for different periods. DCs proteins were extracted with a nuclear and cytoplasmic extraction kit. (**A**) P38, JNK, ERK, and their corresponding phosphorylated proteins were detected by Western blotting with specific antibodies. (**B**–**D**) The quantitative analysis of P-P38/P38, P-ERK/ERK, and P-JNK/JNK protein levels; (**E**) IKKα, IKKβ, IκB, NF-κB p65, and their corresponding phosphorylated proteins in cytoplasm and NF-κB in nucleus were determined by Western blotting. (**F**–**H**) The quantitative analysis of IKKα, IKKβ, P-IKKα/β, IκB, P-IκB, P65, and P-P65 protein levels. Values were normalized to the level of β-actin; (**I**) quantitative analysis of nuclear P65 protein level, values were normalized to the level of histone. The anti-actin antibody and anti-histone antibody were used as the control blots.

As NF-κB and MAPKs downstream pathways are critical in DCs’ activation in a TLR2/4-dependent mechanism, the activation of MAPK (ERK, JNK, and p38) in AEAR-treated DCs was explored in the study (Figure 9A–D). AEAR-TLR2/4-mediated DCs’ activation was found to induce the phosphorylation of JNK, ERK, and p38 at a time manner. The expression levels of ERK and p38 remained unchanged, whereas the basal level of JNK after 45 to 120 min was low. The activation of NF-κB was reported to participate in the phosphorylation and proteolytic degradation of IκB. Therefore, we also detected NF-κB signaling pathways in AEAR-activated DCs (Figure 9E–I). The p-IKKα/β level increased after 10 min, but the total IKKα/β levels remained unchanged. Most of NF-κB subunits remained in the cytoplasm by IκB and translocated to the nucleus after IκB degradation. The degradation of IκB and the enhancement of phosphorylated NF-κB p65 in the cytoplasm after 20 min were time-dependent. AEAR also promoted the nuclear translocation of NF-κB p65. The data showed that ERK, JNK, and NF-κB p38 participated in AEAR-stimulated DCs.

## 4. Discussion

It is necessary to develop new vaccines for emerging diseases such as Ebola and coronavirus disease 2019 (COVID-19). Various kinds of adjuvants are extensively used to elicit an enhanced immune response, thus providing long-lasting protective responses [29,30]. However, most adjuvants are designed based on experiences and related mechanisms are obscure. Therefore, it is difficult to develop novel adjuvants. Many researchers have started to explore new adjuvant options eliciting targeted immune responses in vaccines.

Polysaccharides from TCM have been considered as a promising potential immunostimulator as they can stimulate innate and cellular immunity via the interactions with immunological cell membrane receptors [31,32]. Several compounds have been used as vaccine adjuvants in clinical trials, for example, Advax™ polysaccharide adjuvant, Astragalus polysaccharides adjuvant, ycium barbarum polysaccharides adjuvant, and Ginseng polysaccharides adjuvant [33]. However, whether crude polysaccharides extracted from *Artemisia rupestris* L. could stimulate activation of DCs was unknown. In this study, we firstly prepared the aqueous extracts of *Artemisia rupestris* L. and then found that the main absorption peaks of AEAR in the FT-IR spectra were ascribed to glycosidic structures. AEAR was free of LPS contamination. AEAR could activate DCs through TLR2/TLR4-mediated activation of MAPKs and NF-κB signaling pathways. The blocking of the TLR2/TLR4 pathway inhibited the expression levels of costimulatory molecules on DCs and cytokines. The phosphorylation of important molecules in MAPKs pathway and the nuclear translocation of NF-κB were largely upregulated in AEAR-induced DCs.

The safety of adjuvants is also widely concerned. Although TCM is believed to be effective and safe owing to its long-term use [34], it is still necessary to evaluate the potential toxicity of herbal extracts. As biological response modifiers, immunomodulatory polysaccharides do not cause harm and additional stress on the body, but rather help the body to adapt to environmental and biological stresses. Some polysaccharides from TCM are particularly effective in boosting both Th1/Th2 response with low toxicity and safety, such as Astragalus polysaccharide and Lycium barbarum polysaccharide [33]. In our previous study, we demonstrated that AEAR was a safe and non-toxic adjuvant by acute toxicity tests [15]. In order to further test the potential toxicity and side effects of AEAR, we evaluated the cytotoxicity of AEAR to splenocytes and DCs by cell viability assays and apoptosis analysis for the first time. AEAR in the concentration range of 10 to 1600 μg/mL was not cytotoxic to splenocytes and showed an obvious proliferation activity in a dose-dependent manner. Previous researchers also reported that some plant-derived polysaccharides improved the viability of immune cells [35]. AEAR in the concentration range of 10 to 400 μg/mL was not cytotoxic to DCs, but AEAR with the concentration above 400 μg/mL showed the mild inhibition effect on the growth and apoptosis of DCs. Therefore, AEAR with a concentration below 400 μg/mL was safe in terms of cell viability.

DCs play an important role in the mediation of innate immune response and the induction of adaptive immune response [36,37]. Thus, DCs are applied in vitro platforms to screen novel adjuvants and explore the mechanism of adjuvants. Polysaccharides from *Salvia miltiorrhiza, Ganoderma,* and *Astragalus* [38,39,40] could promote DCs’ maturation. In this study, immature DCs were cultured for 6 d in the presence of GM-CSF and then the cells were observed under an inverted microscope. The observation results indicated that DCs stimulated by AEAR presented obvious mature changes. In fact, AEAR induced DCs’ maturation by up-regulating costimulatory molecules, suggesting that AEAR induced phenotypically mature DCs. The analysis results of microscopy and flow cytometry indicated that AEAR could promote DCs’ maturation.

When DCs are activated, DCs secrete cytokines. It was reported that the enhanced production of IL-1β and IL-6 could promote the immune function of DCs [41]. In the study, we also found that AEAR largely enhanced the production of IL-1β and IL-6 in DCs. Subsequently, we studied the influences of AEAR uptake in DCs maturation on the proliferation of allogeneic T cells and found that AEAR could further promote the effects of DCs’ maturation on the proliferation of T cells and endocytosis of antigens. In our study, we used AEAR to directly stimulate DCs and compared the effects of AEAR and LPS (100 ng/mL). Similar to LPS, AEAR at higher concentrations stimulated DCs’ maturation. These findings suggest that AEAR enhanced the functional and phenotypic maturation of DCs. AEAR had a strong immunomodulation effect on DCs in vitro.

TCM-derived polysaccharides can be recognized by multiple PRRs at the same time, including polysaccharides from *Pleurotus citrinopileatu, Poria cocos,* and *Anoectochilus formosanus* [31,42,43]. Polysaccharides are considered as the key candidates for DCs’ activation without side effects via TLR2 and/or TLR4. The anti-TLR2 and anti-TLR4 antibodies were observed to inhibit DCs’ activation by AEAR and the stimulation effects of AEAR on cytokine production. The analysis results of flow cytometry demonstrated that AEAR could induce DCs’ maturation via TLR2 and TLR4. These data indicated that TLR2 and TLR4 participated in signaling pathways of cytokine production and the expression of costimulatory molecules.

To further explore the mechanisms of AEAR in the activation of DCs after TLR2/4 activation, the downstream events after DCs’ activation were traced. TLRs’ signaling can activate the MAPK pathway, whereas the differential activation of MAPK signaling (ERK, JNK, and p38) can activate transcription factors such as NF-κB [44,45], which in turn regulate the transcriptional activity of a large number of genes in response to immune and inflammatory stimuli. Therefore, the expression levels of key molecules were used to determine whether AEAR activated the downstream NF-κB and MAPK pathways using Western blotting. In this study, we found that AEAR could activate DCs through MAPK signaling pathways, as proved by the phosphorylation states of ERK, p38, and JNK in AEAR-treated DCs. In addition, we also analyzed NF-κB signaling pathways (IKK, IκB, and NF-κB p65). The phosphorylation of IKK and NF-κB p65 was enhanced in DCs and IκB degradation in the cytoplasm and nuclear translocation of NF-κB p65 were also promoted. The changes implied that AEAR activated MAPK and NF-κB through triggering TLR2/4. These signaling pathways may be responsible for the production of cytokines. The above results revealed the molecular mechanisms of AEAR in the activation of murine DCs.

## 5. Conclusions 

In conclusion, AEAR promoted the maturation of DCs without toxicity, stimulated the production of IL-1β and IL-6 and T lymphocyte proliferation, and decreased DCs’ phagocytosis, indicating that AEAR could activate DCs-mediated immune response. TLR2 and TLR4 were responsible for the recognition of AEAR by DCs. AEAR triggered TLR2/4-mediated signaling pathways through phosphorylated ERK, JNK, and p38 and the nuclear translocation of NF-κB. Related signaling pathways and activation effects of DCs induced by AEAR might provide novel insights into the mechanisms of immunomodulation for the application of AEAR in improved vaccines.
